# *‘C’mon, let’s talk:* a pilot study of mental health literacy program for Filipino migrant domestic workers in the United Kingdom

**DOI:** 10.1007/s00127-022-02405-9

**Published:** 2022-12-28

**Authors:** Andrea B. Martinez, Jennifer Y. F. Lau, Hannah Misha Morillo, June S. L. Brown

**Affiliations:** 1 Department of Psychology, Institute of Psychiatry, Psychology and Neuroscience, King’s College London, London, UK; 2 Department of Behavioral Science, College of Arts and Sciences, University of the Philippines Manila, Manila, Philippines; 3 Youth Resilience Unit, Centre for Psychiatry and Mental Health, Wolfson Institute of Population Health, Queen Mary University of London, London, UK; 4 Department of Health Services and Population Research, Institute of Psychiatry, Psychology and Neuroscience, King’s College London, London, UK

**Keywords:** Mental health literacy, Help-seeking, Domestic workers, Migrants, Intervention development, Cultural adaptation

## Abstract

**Purpose:**

This pilot study of a culturally adapted online mental health literacy (MHL) program called *‘Tara, Usap Tayo!’* (C’mon, Let’s Talk) aims to assess the acceptability, appropriateness, feasibility, and potential effectiveness in improving the help-seeking behavior of Filipino migrant domestic workers in the United Kingdom (UK).

**Methods:**

Using mixed methods, we conducted a non-randomized single-group study of the online MHL program with 21 participants. The development of this intervention was guided by the Medical Research Council Framework for developing complex interventions and utilized Heim & Kohrt’s (2019) framework for cultural adaptation. Content materials from the WHO Mental Health Gap Action Program (mhGAP), WHO Problem Management Plus (PM +) and Adult Improving Access to Psychological Therapies (IAPT) were modified and translated into the Filipino language. The MHL program was delivered online in three sessions for two hours each session. Data were collected at three time points: (T1) pretest; (T2) posttest; and (3) follow-up test. Quantitative data on participants’ attitudes towards help-seeking and level of mental health literacy as outcome measures of potential intervention effectiveness were collected at T1, T2 and T3, while focus group discussions (FGDs) to assess participants’ feedback on the acceptability, feasibility, and appropriateness of the online MHL program were conducted immediately at T2. Data analysis was done using a thematic approach for qualitative data from the FGDs and descriptive statistics and repeated-measures ANOVA were used to assess the difference in the T1, T2, and T3 tests*.* Both quantitative and qualitative results were then integrated and triangulated to answer the research questions.

**Results:**

The online MHL program is generally acceptable, appropriate, and feasible for use among Filipino migrant domestic workers. Preliminary findings lend support for its possible effectiveness in improving mental health literacy and help-seeking propensity. The cultural adaptation made in the content, form, and delivery methods of the intervention was acceptable and feasible for this target subcultural group.

**Conclusion:**

By improving their mental health literacy and help-seeking propensity, this online MHL program has the potential to provide support to the mental health and well-being of Filipino migrant domestic workers in the UK. Further feasibility study or large-scale randomized controlled trial is needed to confirm the preliminary findings of this study.

## Introduction

The Covid-19 pandemic has had a disproportionate impact on the physical and mental health of ethnic minorities in the United Kingdom (UK) [1]. Hidden within these communities are Filipino migrant domestic workers with low-paid jobs, many of whom are victims of human trafficking and modern-day slavery. Migrant domestic workers constitute one of the largest migrant populations in the world [2] and are among the most vulnerable migrant workers. They work for private households [3] as nannies, cleaners, chauffeurs, cooks, private carers, gardeners, or drivers [4]. They are considered ‘invisible’ [9] and ‘expandable’ yet ‘essential’ [5]. They work in jobs considered dirty, dangerous, difficult, and degrading [6]. They are also usually tied to their employer through a regulated work permit [2]. The International Labour Office (ILO) estimates that there are around 11.5 million migrant domestic workers in the world and around 74% [3] to 98% [7] of them are women, making it a highly feminized labor sector [8]. The Philippines is the top supplier country of migrant domestic workers in the world, followed by Indonesia and Sri Lanka [9] In the UK, more than half of the 20,000 domestic workers’ visas issued yearly are for Filipino migrant domestic workers [10].

The precarity of living away from their families and working in private households [12] makes them prone to isolation and loneliness, anxiety, and trauma, and hence vulnerable to exploitative work conditions [13]. The effects of the Covid-19 lockdown further exacerbated their already vulnerable situation and like other ethnic minorities in the UK [14], many of them have experienced joblessness, destitution, and subsequent mental health problems.

However, studies have shown that people from culturally diverse backgrounds such as migrant domestic workers underutilize mental health services [15, 16], seek professional help only in severe cases [17], and are most likely to drop out of treatment because of cultural differences with the mental health specialist [18]. Other cultural and structural barriers that hampered their help-seeking include language difficulty, misconceptions, a sense of shame and stigma on mental health, restricted immigration status, lack of awareness of rights, and uncertainty about the availability of services [19]. There is also a sense of cultural mistrust and experience of discrimination by service providers from a different ethnic group and/or cultural background [20], contributing to reduced tendencies to navigate available services and resources. These barriers are seldom addressed since most accessibility programs for mental health care are focused on the ‘supply side’ of the healthcare system such as improvement in the availability and quality of treatments, with less emphasis given to problems associated with accessing these resources and services [21].

Research has also shown the widening gap between the prevalence of mental disorders and the availability of mental health services [22]. It is estimated that the treatment gap for common mental disorders is 60% in high-income countries, 65% in upper-middle-income countries and over 80% in lower-middle-income countries [23]. Thus, there is a need to develop evidence-based interventions that can reach as many people as possible whilst taking into account the cultural and contextual factors of different subgroups [24]. To address the treatment gap, the World Health Organization (WHO) [25] and the Lancet Commission on Global Mental Health and Sustainable Development [26] recommend scaling up evidence-based psychological interventions which usually have been developed in higher-income countries. This means expanding the ability of interventions that are efficacious on a small scale and under controlled conditions to be useful to a larger proportion of the population in real-world settings while retaining effectiveness [27]. Low-intensity interventions (i.e., delivered by trained non-specialists or in self-help formats) that are brief, and easy to understand are potentially scalable [28]. This includes mental health literacy program (MHL) which has been recommended for underserved populations like migrants, refugees and asylum seekers [29].

In recent years, “mental health literacy” (also called mental health awareness) has been significantly expanded to include not just knowledge and beliefs about mental disorders but also skills in recognizing, managing, and preventing mental health problems [30]. It now includes increasing awareness of help-seeking options and available professional care for mental disorders and skills such as self-help strategies to manage mild symptoms or maintain positive mental health. Mental health literacy (or MHL) programs also include mental health first aid to support others with mental health problems and more generally, aim to reduce the stigma associated with mental disorders [31]. Research has demonstrated that increasing engagement in MHL programs even briefly may improve the recognition, management, and prevention of mental health problems [32]. This approach is appropriate for Filipino migrant domestic workers facing structural and cultural barriers around accessing services. There is also a need to suit intervention to the culture, needs and expectations of target subcultural groups to enhance effectiveness and acceptability [20, 33].

Cultural adaptation is defined as “the systematic modification of an evidence-based treatment or intervention protocol to consider language, culture, and the context in such a way that it is compatible with the client’s cultural patterns, meanings, and values” [34]. Cultural adaptation aids in reducing inequities in mental health care especially among migrant populations by adapting evidence-based interventions to their diverse needs and cultural backgrounds [35]. More specifically, by integrating social circumstances and cultural factors such as values, beliefs and language into mental health interventions, the relevance, acceptability, and effectiveness of treatments will be improved [33]. Culturally appropriate interventions delivered in a community-based setting or within the perceived safe environments of familiar community organizations have also been shown to achieve better outcomes [36].

This pilot study describes a new culturally adapted mental health literacy (MHL) program called ‘*Tara, Usap Tayo’* (C’mon, Let’s Talk). We aimed to assess its acceptability, feasibility, appropriateness, and potential effectiveness in improving mental health literacy, help-seeking behaviors, and coping strategies of Filipino migrant domestic workers in the UK.

## Materials and methods

### Study design and selection of participants

#### Participants

The development of the intervention targets Filipino migrant domestic workers in the UK as one of the most vulnerable groups of migrants who underutilize mental health services [37]. As the target subcultural group for a larger intervention study, this pilot study was conducted with 21 female Filipino migrant domestic workers in London, based on the recommended minimum sample size of 12 for pilot studies [38]. Participants were recruited through various community-based Filipino organizations in the UK. Recruitment advertisements with links to sign-up sheets were posted on the social media accounts of these organizations after getting consent from their officials. Selection criteria included Filipino migrant domestic workers in the UK, regardless of immigration status or gender. However, we excluded those who recently experienced extreme trauma and psychological distress because it might be too risky for them to participate in break-out sessions where sharing of immigrant experiences was expected, and they might display negative emotional reactions; this was screened at sign-up [39]. Although none of the participants has recently experienced trauma, one had recently lost her partner for 10 years due to Covid-19. She was given the autonomy to decide if she still wanted to participate. Participants were contacted through emails and/or mobile numbers they provided in the sign-up sheet, together with the schedule and Zoom link for the sessions. Individuals who signified their intention to join and passed the screening questions were sent the Information Sheet (translated into Filipino) about the nature and objectives of the study. Online informed consent (also translated into Filipino) was sought during the first session. Participation was voluntary. To protect anonymity, participants were advised to use pseudonyms in answering assessment questions and in the focus group discussions. These were also used in reporting the results. Personal identifying information was also removed and only their pseudonyms and socio-demographic information reported as group data such as age group, work and immigration status were retained. Ten of the participants are working full-time, six are working part-time, four have recently lost their jobs and are actively seeking work and one at the time of the study cannot work because of childcare. They have been in the UK for an average of 8 years. Only four had a valid work or residence visa, seven are on appeal of their asylum cases, and ten are overstaying. They performed a variety of work, from all-around domestic work to cleaning and housekeeping, acting as a nanny, personal assistant, or health and social carer. To ensure that sessions were manageable with a smaller number of participants, two groups were formed: 10 participants for the first group and 11 for the second group.

#### Design

Using mixed methods, we conducted a non-randomized single-group case series study design with three time points: (T1) pretest; (T2) posttest; and (3) follow-up test. We incorporated the use of self-report questionnaires with focus group discussions (FGDs) to address the key questions of feasibility and acceptability of the intervention and self-report questionnaires as measures of the potential effectiveness of the intervention. Quantitative data on participants’ attitudes towards help-seeking and level of mental health literacy as outcome measures of potential intervention effectiveness were collected at T1, T2 and T3, while focus group discussions to assess participants’ feedback on the acceptability, feasibility, and appropriateness of the MHL program were conducted immediately at T2. T1 was administered a week before the first psychoeducation session and T2 was done immediately a week after the last psychoeducation session. T3 was conducted two months after the posttest to check the retention of attitude change. To minimize participant bias, a research assistant administered the assessment tools and facilitated the FGDs. A post-intervention focus group discussion was chosen as the research method as it serves both functions of dialog and interaction [40] among participants with the assumption that they have achieved a certain level of comfort and negotiated interaction with each other after their engagement in the MHL intervention program. The use of break-out rooms and small group discussions where participants freely shared ideas and experiences was aimed at facilitating rapport and comfort among them. Participants were reimbursed £10 each for their time and mobile data usage after completion of the assessment at each time point. Ethics clearance was obtained from the university’s Institutional Review Board (Ref. No. HR-19/20-17,175) which approved all communications, information sheets, consent forms, and methods used in this research.

#### Measures

A semi-structured interview guide adapted from Saracutu et al. protocol [39] was used in the FGD to assess the acceptability, appropriateness, and feasibility of the MHL program and triangulate its potential effectiveness (Table 1). We defined acceptability as the participants’ report of agreeableness and satisfaction in taking part in the MHL program, appropriateness as the participant’s perception of the relevance and good fit of the intervention into their needs and situation as migrant domestic workers, and feasibility based on the participants’ retention rate and reports of the barriers they encountered in participating in the intervention [41]. Three self-report questionnaires in digital format and translated into the Filipino language (while retaining the English version) were administered at different time points to assess their potential effectiveness: (1) Inventory of Attitudes towards Seeking Mental Health Services (IASMHS); adapted versions of (2) Mental Health Literacy Scale (MHLS); and (3) General Help-Seeking Questionnaire (GHSQ).

**Table 1:** Semi-structured Interview Guide

Outcome measures	Questionnaire
Acceptability and feasibility	How would you describe your experience of taking part in *Tara, Usap Tayo* (C’mon, Let’s Talk about Mental Health) program?
Process of change and potential effectiveness	What did you learn from participating in the MHL program?
Acceptability	What aspect of the program did you like the most or was easier for you to understand? What is your favorite activity (or topic)?
Acceptability and feasibility	What did you least like about the MHL program or which part was difficult for you to understand? What do you think could be improved about *Tara, Usap Tayo* (C’mon, Let’s Talk about Mental Health) program?
Feasibility	What difficulties and/or barriers have you encountered in taking part in the MHL program?
Process of change and potential effectiveness	What have you noticed are the differences in your attitude and knowledge about mental health as a result of taking part in *Tara, Usap Tayo* (C’mon, Let’s Talk about Mental Health)?
Appropriateness	How suitable is the MHL program to your needs and to your situation?
Acceptability	Would you recommend this intervention to others?

The MHLS is a 35-item questionnaire that is used to assess knowledge of mental health information including symptoms of common mental health problems and available mental health services. For this study, specific items on mental health problems (e.g., bipolar disorder, schizophrenia, etc.) and psychotherapy (i.e., cognitive behavior therapy) were omitted and only the remaining 24 items on help-seeking were administered to participants as a form of adaptation of the scale [42] to the contents and objectives of the current MHL program. These items are deemed very Westernized and not within the scope of Filipino linguistic concepts of mental health, such as schizophrenia, bipolar disorder and cognitive behavior therapy [43]**.** Studies have also shown that lay people poorly recognize labels of mental health disorders [44, 45], with Asians less familiar with these terms than Westerners [46]. These mental disorders are also not targets of the MHL intervention program because it is more focused on anxiety and depression as common mental health disorders. Higher total scores in the MHLS indicate a high level of mental health literacy. The MHLS has reliability measures of alpha 0.797 [47].

The IASMHS has 24 items that measure attitudes toward mental health help-seeking, with three subscales on psychological openness (defined as an acknowledgement of psychological problems and seeking professional help), help-seeking propensity (willingness and ability to seek professional services), and indifference to stigma (concerns about other people’s perception about their help-seeking for their mental health problems). Items are scored from 0 (disagree) to 4 (agree) and levels of a positive attitude are determined by getting the total of all the items, with some items scored in reverse. Psychometric properties of IASMHS show a full-scale alpha of 0.87 in all items, 0.82 for the Openness subscale, 0.76 for Help-seeking Propensity and 0.79 for Indifference to Stigma [48].

The GHSQ is used to measure the likelihood of seeking help from various sources (e.g., spouse, friend, general practitioner, mental health specialist, religious leader, etc.) when individuals experience personal distress and/or suicidal ideation. It uses a 7-point Likert rating scale which ranges from 1 (extremely unlikely) to 7 (extremely likely) for each source of help. In this study, the following sources of help were included in the GHSQ items in addition to the original version: children, charity organizations, employers, and colleagues. The scale reliability of GHSQ shows a Cronbach’s alpha of 0.85 [49].

### Description of the intervention

#### Development of the intervention

The MHL program, *‘Tara, Usap Tayo’* (C’mon, Let’s Talk) is brief non-specialized support in the form of psychoeducation program that was culturally adapted from the contents of the WHO Mental Health Gap Action Program (mhGAP) Intervention Guide [50], WHO Problem Management Plus (PM +) [51] and Adult Improving Access to Psychological Therapies (IAPT) of the UK National Institute for Health and Care Excellence [52] to address Filipino migrant domestic workers’ mental health needs and patterns of help-seeking behavior. Using an evidence-based approach, the development of this MHL program followed the guidance of the Medical Research Framework (MRC) for developing complex intervention, which stipulates four key phases of research, namely: (1) development or identification of intervention; (2) feasibility; (3) evaluation; and (4) implementation [53]. As recommended by the MRC Framework [54], a systemic review was conducted to assess the evidence [19], followed by qualitative research using key informant interviews with Filipino migrant domestic workers [37] as stakeholders to inform the development of the intervention. Questions on what constitutes a culturally appropriate and acceptable mental health intervention as part of further refinement of the intervention were probed during the qualitative study phase. Narratives shared by participants and anonymized in the qualitative phase also provided relevant and culturally appropriate case examples that were adapted in the current intervention program.

#### Cultural adaptation of the intervention

The cultural adaptation of the MHL intervention followed the framework proposed by Heim and Kohrt [28] which considers three elements: (1) cultural concepts of distress; (2) treatment components; and (3) treatment delivery. The first adaptation component includes cultural explanations and expressions of distress. The second adaptation component includes specific (e.g., interpersonal, and emotional) and non-specific (e.g., discussion on the benefits of intervention) therapeutic elements [55] as well as in-session techniques; the latter should consider whether they are acceptable and accessible to the target subcultural group [56]. To improve intervention engagement, Heim and Kohrt [28] recommend providing explanatory models that are culturally congruent to the beliefs, values, and practices of the target population. For example, adaptation should address culturally specific notions of stigma and how they affect the help-seeking behaviors of people with mental health problems due to fears of stigmatization [57]. The third adaptation component should take into account the appropriateness of treatment modalities to the socio-demographic and cultural backgrounds of the target subcultural group. For instance, it should consider whether using delivery formats and materials such as face-to-face or online interventions, group-based or self-help [58], language and metaphors, or case examples are fitting to the target population. Heim and Kohrt’s framework [28] are potentially applicable to low-intensity (i.e., no contact with a therapist), standardized evidenced-based treatments such as guided or unguided self-help format or alternatively, those delivered by trained lay persons. Hence, it has the potential for scaling up interventions [25].

Content materials from the WHO mhGAP, WHO PM + and IAPT were modified and translated into the Filipino language. Filipino cultural concepts that pervade participants’ understanding of mental health and *‘ginhawa’* (well-being) were integrated into the MHL program as an explanatory framework for mental health in addition to the WHO definition [50] (Table 2). For instance, Filipino migrant domestic workers’ beliefs on the dichotomy of mental health (e.g., soundness of mind) and illness (e.g., having a ‘troubled mind’) were integrated with the explanations on the spectrum of mental well-being [59]. Because mental health issues are believed to be caused by difficult and challenging life experiences, their preferred sources of help are informal networks of friends and relatives who can provide adequate social support [37]. This was considered in the cultural adaptation of the contents on addressing barriers in help-seeking and in the discussion of the referral pathways to mental health services in a way that is culturally congruent with their beliefs. In addition, the toughness of character and resilience as valued traits for Filipino migrants were highlighted through discussions on self-care. Together with other barriers to help-seeking such as lack of familiarity with health services, stigma tolerance and cultural mistrust, they were incorporated as discussion points on the benefits of the intervention as a non-specific therapeutic element of the second adaptation component. The third adaptation component of treatment delivery includes the use of culturally relevant case studies and examples to emphasize key concepts; they incorporated Filipino migrants’ narratives and experiences. Although the MHL program was designed using a face-to-face format, the Covid-19 lockdown restrictions in the UK necessitated online delivery of the intervention and an adaptation of the materials into a digital format. Both the psychoeducation and the FGD were delivered and facilitated entirely in the Filipino language by the first and third authors. Scaling up low-intensity interventions such as the WHO’s mhGAP and PM + also requires the training of a pool of non-specialists to provide mental health services in the community. In view of this goal, the MHL program also included the facilitation of skills-building exercises among participants on how to identify symptoms of common mental health problems and understand different referral pathways to mental health services so they can be trained as laypersons delivering community mental health support in the future.

**Table 2:** Filipino cultural concepts and their adaptation

Filipino cultural concepts of well-being	Cultural symbolisms and meanings	Cultural adaptation in the study
*Tara, usap tayo!*	Loosely translated as ‘C’mon, Let’s Talk!’ This is a common catchphrase in the Philippines for a sensitive topic requiring serious conversation	This has been used as the title of the mental health literacy program
*Ginhawa/ Kaginhawaan*	State of complete well-being or wellness, consists of 4 dimensions: *kalooban, kapwa, kakayanan* and *kabuluhan*	Used in framing the discussion on mental health and well-being
*Kalooban*	Inner reality/being consists of positive and negative changes in thoughts, feelings and behavior	Used in framing the discussion on mental health and well-being
*Kapwa*	External reality and shared identity consist of adaptive and maladaptive changes in our relationship with the family, work and environment	Used in framing the discussion on mental health and well-being
*Kakayanan*	Empowerment comes from our ability to recognize and use our own resources as well as the availability of external support	Used in framing the discussion on mental health and well-being
*Kabuluhan*	Peace of mind, the meaning of the life; refers to our state of inner peace or our ability to give positive meaning to adverse events	Used in framing the discussion on mental health and well-being
*Tibay at tatag*	Strength and endurance of character; approximates resilience	Used in framing the discussion on self-care and resilience
*Kaagapay/Kabalikat*	Approximates the role of constant and reliable companion/friend/ally	Used in framing the discussion on help-seeking, seeking support
*Bamboo as a metaphor for resilience*	The bamboo is as a symbol of Filipino resilience, it does not break but only bends in the direction of the wind	Used in framing the discussion on self-care and resilience
*Buntong-hininga*	Deep breathing	Used as a relaxation technique

#### Description of the sessions

Three weekly sessions (two hours each) of the MHL program (Table 3) were conducted online using the Zoom platform. Participants used their mobile phones to join the sessions, and only one used a laptop. A total of six sessions per group of participants were completed, three of which were for psychoeducation and the other three for the administration of assessment tools and facilitation of the FGD. These sessions were done on five consecutive Sundays during days off from work; the sixth session (done two months after the T2) was also on Sunday. To protect anonymity, participants were reminded both in the Information Sheet and at the start of each session that they can turn off their video and use pseudonyms they indicated in answering the assessment test and informed consent forms. Each session was divided into two topic presentations of 20–25 min each and punctuated by two break-out sessions of 15–20 min each for small group sharing. The topics covered in the MHL program included the following: (1) explanatory models of mental health and illness incorporating the *ginhawa* (well-being) framework; (2) recognizing signs and symptoms of depression and anxiety as common mental health disorders; (3) overcoming barriers to help-seeking; and (4) self-care and developing resilience. Short video films produced by Maudsley Learning Online on mental health (https://www.youtube.com/watch?v=G0zJGDokyWQ) and by the WHO on depression (https://www.youtube.com/watch?v=PYbuB-Ateus) that were accessible online were used to summarize the discussion and each session ended with the participants’ ‘primary take-away’ lessons. Participants were given a soft copy of the presentation materials for personal reference.

**Table 3:** Psychoeducation topics in the MHL program

Time allocated	Topics
First psychoeducation session	
10 min	Welcome and ground rules
15 min	Self-introduction and expectation setting
5 min	Busting myths on mental health and mental illness
20 min	Basics of mental health: definition, global burden of disease, the mental health spectrum, the biopsychosocial framework and Filipino cultural model of well-being (the ‘Ginahwa’ or ‘well-being’ framework`), importance of mental health
15 min	Break-out room: *Migrants’ experience of mental health well-being and mental ill-health using Filipino ‘ginhawa’ (well-being) framework*
5 min	Plenary synthesis of group sharing
20 min	Basics of mental ill-health: definition, stress vs. traumatic stress, risk factors, the mental health impact of the Covid-19 pandemic, mental health stigma
15 min	Break-out room: *Covid-19 lockdown: How the pandemic affected one’s well-being?*
5 min	Plenary synthesis of group sharing
10 min	Summary, short film on ‘What is Mental Health’ produced by Maudsley Learning Online, ‘take-away lesson’ from each participant
Second psychoeducation session	
Time allocated	Topics
15 min	Welcome, reminders of ground rules, recap of the previous session
5 min	Busting myths on anxiety and depression
20 min	Basics of depression: warning signs, risk factors, prevalence, prevention, positive coping strategies, and self-care
20 min	Break-out room: *It’s Okay Not to Be Okay: Recognizing signs of depression*
5 min	Plenary synthesis of group sharing
20 min	Basics of anxiety: warning signs, risk factors, prevalence, prevention, positive coping strategies, and self-care
20 min	Break-out room: *It’s Okay Not to Be Okay: Recognizing signs of anxiety*
5 min	Plenary synthesis of group sharing
10 min	Summary, short film on depression (Let’s Talk: Angelo’s Story) produced by WHO Regional Office for the Western Pacific, ‘take-away lesson’ from each participant
Third psychoeducation session	
Time allocated	Topics
15 min	Welcome, reminders of ground rrules, a recap of the previous session
15 min	Common barriers to mental health help-seeking
15 min	Break-out room: *Unloading my stress container: Whom and how to seek help?*
5 min	Plenary synthesis of group sharing
15 min	*Kaagapay at kabalikat:* Accessing mental health services in the UK and understanding referral pathways
15 min	Activity: *Simulation exercise on accessing mental health services in the UK*
5 min	Plenary synthesis of group sharing
15 min	*Tibay at Tatag* (Strength and Endurance): Developing individual and community resilience, self-care
10 min	Activity: *My pledge of commitment to self-care*
10 min	Summary, ‘take-away lesson’

### Data analysis

Qualitative data from the FGDs were analyzed using a thematic approach through NVivo 12 software program. Two FGDs were conducted in the Filipino language and audio recorded. Transcripts were transcribed verbatim and then translated from Filipino into English languages. These transcripts were read several times and exemplary quotes that are relevant to the purpose of the study were condensed into meaningful units. They were then tabulated, coded, and then sorted into themes and sub-themes. Quantitative data were analyzed using IBM SPSS Statistics (Version 23) predictive analytics software. Repeated measures ANOVA was used to assess the difference in T1, T2 and T3 tests while effect sizes were computed using Pearson’s correlation coefficient *r.* Both quantitative and qualitative results were then integrated and triangulated to answer the research questions pertinent to the acceptability, feasibility, appropriateness, and potential effectiveness of the online MHL program.

## Results

### Acceptability, appropriateness, and feasibility of the online MHL program

Participants’ thematic responses on the acceptability, appropriateness, and feasibility of the online MHL program can be grouped into three categories: (1) relevance to their needs and situation as migrant domestic workers; (2) positive group experience and expansion of social networks; and (3) feasibility issues and problems encountered.

All participants affirmed that the online MHL program is useful and appropriate to their needs and situation as migrant domestic workers who are often isolated and prone to stress. The sessions gave them an opportunity to interact with each other. For example, Iza explained why the intervention is fitting to her needs, *“especially in my work situation where I must work from Monday until Saturday, sometimes even on a Sunday, I’m feeling all the stress especially that it’s lockdown and my bosses are working from home… So, I look forward to a Sunday when we have an online seminar, coz it gives us a chance to share our experiences and it gives us time to listen to others’ experiences as well. So, for me, this is very suitable, and it helps me immensely.”* The online MHL also served aa s breather from the drudgery and monotony of engaging in domestic work in private homes during times of lockdown. Joy pointed out how helpful the sessions are for her, saying, *“Especially for domestic workers like us who are exhausted from work every day, physically we’re already tired, but does our mental health also have to get exhausted?. I really need this one. it’s very helpful.”* For others who recently experienced distress, the online MHL program taught them how to bounce back, like Mar who said, *“It’s very appropriate because it resonates especially with me that I just came out of depression last month… it’s such a big help for me… that we are talking about things like this (mental health)… especially for those like me who are going through difficult times.”* As migrants, they bear the added responsibility of being burdened by family problems in the Philippines. As such, the sessions were also helpful for them, as explained by Vee, *“this is great, especially for us who are far away from our families, we are prone to (experiencing) loneliness. We also bear the problems of our families in the Philippines and on top of that we also have our problems here at work with our employers, that’s why we really need this kind of seminar because it helps.”*

The content, form, and method of delivery of the online MHL program were reported by participants to be acceptable as indicated by their favorable attitude towards mental health topics covered in the discussion and their interest and enthusiasm in engaging in various group activities during the sessions. Participants reported having a positive experience of group sharing during break-out room sessions. Mik described her experience as feeling comfortable and trusting, *"In our breakout room, we immediately developed trust and confidence. I had the realization that even if I do not know these people well, I can easily open myself to them*. *I can relate to what most of them are saying.*” For many of them, they found commonality in their experiences by discussing them in the context of small groups. They described it as *“a form of relief,” and “learning from and caring for one another.”* Lei said, *“I like discussing with the group my own experience of depression*. *As they say, sharing is caring.”* They said that they will recommend the online MHL program to others. According to Nie, it was *“educational, helpful, supportive and applicable.”* She said it is educational because she learned several concepts and ideas, such as *“differentiating between mental health and mental illness.”* The online MHL is also useful *“…because we can use it in our lives… we learned where we could go for help.”* It is also applicable for them as migrant domestic workers *“because we can apply things we learned in our lives.”*

On the other hand, participants reported a range of problems and challenges they encountered in joining the online MHL program, including problems with time availability and managing schedules, as well as their internet connections. For instance, Ella said, *“it was difficult coz the following morning I have an early work schedule,”* whilst Len had problems with conflicting schedules, saying, *“there were times when there was simultaneous online seminar (schedules)… then there were times that even on a Sunday, I have a job. So, I have problems with scheduling. I need to identify my priorities.”* They also found it difficult to engage in the assessment tasks, describing them as *“confusing,” “lengthy”* and *“taking tests under pressure adds to our anxiety.”* Despite this, the retention rate was high with almost 97 per cent attendance in all three sessions; only two participants missed one session each.

### Potential effectiveness of the online MHL program

When probed on the potential effectiveness of the intervention, five themes emerged from the FGDs when participants talked about their experience of the online MHL program: (1) increased understanding of mental health and the signs and symptoms of mental health problems; (2) recognition of different forms of help-seeking; (3) increased stigma tolerance and reduced self-stigmatizing beliefs; and (4) developing positive coping and learning about the importance of self-care and self-help skills as preventive measures of mental health problems. These themes are summarized in Table 4 together with the themes on acceptability issues.

**Table 4:** Summary of qualitative themes

Acceptability, appropriateness, and feasibility of the online MHL program
(1) relevance to their various needs and situation as migrant domestic workers
(2) positive group experience and expansion of social networks
(3) feasibility issues and problems encountered
Potential effectiveness of the online MHL program
(1) increased understanding of mental health and the signs and symptoms of mental health problems
(2) recognition of different forms of help-seeking
(3) increased stigma tolerance and reduced self-stigmatizing beliefs
(4) developing positive coping and learning about the importance of self-care and self-help skills

**Fig. 1:**
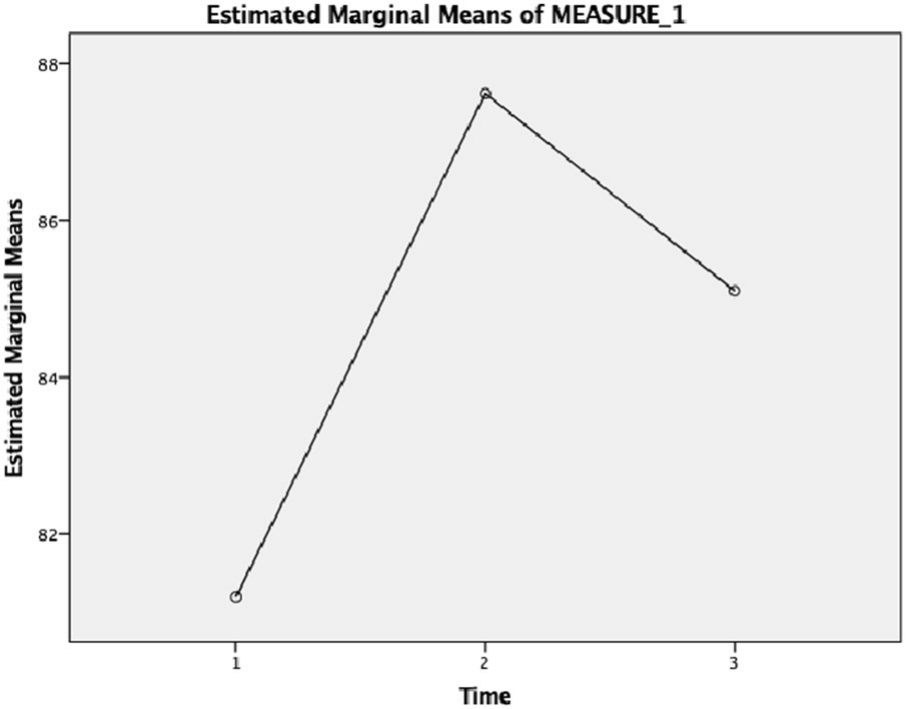
Levels of mental health literacy

Results of the qualitative data from the FGD showed an improved understanding of the difference between mental health and mental illness, the signs and symptoms, and risk factors associated with common mental disorders such as anxiety and depression. For instance, Nie said, *“I cannot forget (what I learned) about the physical manifestations of stress… when I feel shoulder pain, muscle tension, I realized that they can be signs of stress. I thought I was just overworked. Or when you cannot sleep at night, it can be a sign of anxiety or depression.”* This was supported by quantitative data in their MHLS scores. A repeated-measures ANOVA showed a statistically significant increase in participants’ levels of mental health literacy (F (2, 40) = 14.524, *p* = 0.000), an increase that was further maintained at the two-month follow-up in three time points: T1 (*M* = 81.19, SD = 8.583), T2 (*M* = 87.62, SD = 9.119) and T3 (*M* = 85.10*, *SD = 7.842). See Fig. 1 for an illustration of participants’ significant improvement in their mental health literacy.

Participants also expressed a positive change in their attitude towards seeking help from various sources and other help options available when experiencing distress. They have a recognition of the importance of formal help such as Vee who shared, “*if you have symptoms of anxiety or depression, you should not be embarrassed to ask for help, especially from a specialist.”* On the other hand, most participants still valued informal sources of help such as friends and community organizations. Ara said that it is important to have an *“organization that you can come to at a time when you are having depression”* and “*a friend whom you can share your experience with so that they can help… when you are going through difficult times.”*

**Fig. 2:**
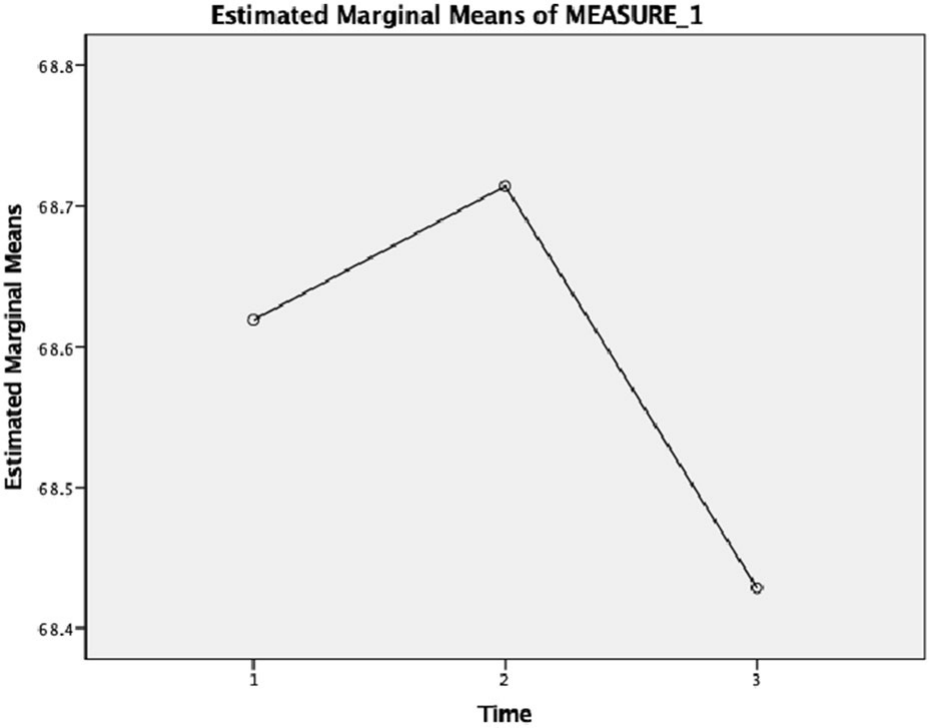
Attitude towards mental health help-seeking

This change in help-seeking attitude is reflected in the quantitative data on their general help-seeking behavior. Participants indicated that their top three choices for sources of help when experiencing emotional distress are the following: in T1 are parents, charity organizations, and siblings; in T2 are charity organizations, parents, and professionals; and in T3 are general practitioners, parents, and professionals. This trend would indicate an increased willingness to seek professional help. When having suicidal ideation, their top three choices for sources of help are: in T1 are professionals, charity organizations, and parents; in T2 are professionals, GP, and charity organizations; and in T3 are GP, professionals, helpline and priests or clergy. However, in measures of attitudes towards help-seeking, a repeated-measures ANOVA showed no significant overall effect of time (F (2, 40) = 0.006, *p* = 0.994), given their average scores in three time points: T1 (*M* = 68.62, SD = 9.415), T2 (*M* = 68.71, SD = 10.729) and T3, (*M* = 68.43, SD = 10.562). When examining subscales, there was a significant increase in help-seeking propensity (or their willingness and ability to seek professional services) **(**F (2, 40) = 4.239, *p* = 0.021), but no significant changes emerged on other subscales of psychological openness (or their acknowledgement of their psychological problems and need for professional help) and indifference to stigma (or their concern about people’s perception about their mental health help-seeking). Figure 2 shows the level of their attitudes towards mental health help-seeking at three different time points in the study.

The online MHL program also helped in destigmatizing mental illness and debunking cultural myths that prevent Filipino migrant domestic workers from seeking help. The participants reported positive changes in their attitude towards people with mental health problems from being dangerous to needing sympathy and understanding, indicating an increased level of stigma tolerance. Len said *“that a friend with depression or mental health problem should not be avoided or feared… because we always have this impression that when we see someone who is a bit crazy, we feared the person. But after this online seminar, I learned that we should not have that kind of reaction, that we should not fear them because they are more vulnerable than us.”* They also reduced their self-stigmatizing beliefs which led to their recognition of the importance of help-seeking and talking about their mental health issues. Ella said *“it is not a shame to experience anxiety or depression. Before, I usually just keep my feelings bottled up inside me because I was embarrassed to ask for help from anyone because people might think that I’m getting crazy.”*

Participants developed a range of self-help and positive coping strategies for the prevention and management of mild distress. Iza said she now knows *“how to build personal resilience and how to relieve anxiety or depression*” by *“calming methods and other exercises.”* According to Beth, *“the simple tips I learned from the webinar like deep breathing, or walking (and being active) when you have depression, they are very important for me because I’m able to use them in my life.”* Participants also learned self-care, such as Joy who said *“I really like (the idea) of ‘self-care is self-love and self-compassion,’ I’m already applying that to myself.”* Others reported increased self-confidence, as to how Eva described her experience that *“it is self-empowering.”* Participation in the online MHL program also allowed them to enhance their self-awareness such as Iza who gained valuable knowledge about herself such as *“when I feel that I’m getting anxious, I can tell myself that I might need help because I’m already having a moderate level of anxiety.”*

As their take away lessons, Ara said, *“what sticks in my mind is the importance of taking care of our mental health, that we should not just take care of our physical (health), but that we should also take care of our mental (health).”* She likened mental health problems to physical health issues, saying, *“When we have slight headaches, we immediately take medications, so for our mental health, if we (experience) mental illness, we must do something to prevent it from getting worse.”* Joy re-affirmed this, saying, *“it’s not just our physical body that gets tired, our mind also gets exhausted.”*

## Discussion

The results of this pilot study indicate that a culturally adapted online MHL program may be an acceptable, appropriate, and feasible intervention amongst ethnic minorities like Filipino migrant domestic workers. Although technical problems and time constraints have been considered feasibility issues, participants affirmed that the contents, form, and methods of delivery of the online MHL program are appropriate to their needs and situation as migrant domestic workers. Results also indicate its potential effectiveness as significant improvements were reported in participants’ mental health literacy and help-seeking behaviors following their engagement in the online MHL program. There was a significant increase in their baseline levels of knowledge of mental health literacy that lasted until two months post-intervention. This was supported by their qualitative reports of how they developed a greater understanding of mental health issues using the Filipino *‘ginhawa’* (well-being) framework, and subsequently their help-seeking behavior. This indicates the importance of adapting the intervention to the culture, needs and expectations of target subcultural groups such as Filipino migrant domestic workers to enhance its effectiveness and acceptability as different cultural groups have different expressions [60] and explanations [61] of symptoms of mental illness which influence their help-seeking and treatment choices.

There were also some trends found in their attitudes to help-seeking. Although no significant change is noted in participants’ general attitude towards help-seeking in quantitative measures, specifically on their psychological openness and indifference to stigma, it does not necessarily mean that the intervention has no impact on their formal help-seeking attitude. The lack of significant difference in these two sub-scales might be due to the small sample size (*n* = 21), which means that it is underpowered to detect any significant change. Their propensity to seek help from professionals was significantly increased until follow-up assessment as indicated in their scores in their MHLS and GHQ tests and their preference for sources of help shifting from informal (e.g., parents and other family members) to formal help (e.g., charity organizations, GPs, professionals, and helplines). This was supported by the thematic results in the FGDs showing increased stigma tolerance and reduction of self-stigmatizing beliefs that led to their recognition of the importance of help-seeking.

### Acceptability and appropriateness of the culturally adapted online MHL program

As migrant domestic workers who usually work in private homes and live with their employers, they are at a disadvantage due to limited social interaction and opportunities for open conversations about mental health issues. The online MHL program as a targeted intervention is seen as acceptable and beneficial to this under-represented population group. Their active engagement in group sharing is an indication that they are ready to talk about their personal experiences in a safe space where the discussion of sensitive topics like mental ill-health is normalized. This helps them vent out pent-up emotions that they would not likely share with others if they felt discriminated against or stigmatized, thus getting the much-needed social support from friends and peers [62]**.** Participants’ favorable attitude towards the intervention is also evident in their enthusiasm to share what they learned. They consider the online MHL program worth recommending to family and friends.

Filipino migrant domestic workers also perceived the intervention as relevant and tailored to their psychosocial and cultural needs, thus increasing the acceptability and appropriateness of this culturally adapted intervention. They appreciated the integration of the Filipino cultural concept of *ginhawa* (well-being) and how its four dimensions relate to their concept of distress: (1) *kalooban* (inner reality—or how their thought, feelings and behavior are affected by their psychological distress); (2) *kapwa* (external reality and shared identity—or how their relationship with significant others are affected by distress but can be a source of strength); (3) *kakayanan* (sense of empowerment—or what internal and external resources are available to help them cope); and (4) *kabuluhan* (inner peace or sense of meaning—or what significance, life lesson or positive meaning do they attach to their distressing experience). They agreed that the material was easily understandable, and the online MHL program fulfilled their expectations. The use of the native language, the metaphor of the bamboo as a symbol of Filipino resilience, and the application of case examples relevant to their migration experience was useful in delivering and highlighting the key messages of the mental health literacy program. For a culturally adaptive intervention program to be acceptable and appropriate, it is imperative to consider the cultural and linguistic diversity of its target community [30]. Evidence suggests that using the local language of participants is an effective way to get positive outcomes from the intervention [28]. In addition, the facilitation of role-playing and simulation exercises of *kaagapay/kabalikat* (reliable companion) in the online MHL program was helpful in developing their skills and competence in accessing mental health services and finding referral pathways. This is consistent with the Filipino social norm of having an intermediary or companion when navigating an unfamiliar domain. Research evidence has established that adaptation components that are congruent with the life situation, experiences and cultural values of the target subcultural group are more likely to be accepted [63].

### Feasibility of the culturally adapted online MHL program

Difficulties reported by migrant domestic workers in their engagement in the online MHL program were mostly focused on their limitations of using a remote or online activity such as poor internet connection, technology issues, and time constraints. However, certain benefits of an online mode of psychoeducation include cost-saving, convenience [64, 65], and flexibility in terms of scheduling, and thus can be structured around participants’ availability. Studies have shown that when barriers in treatment are addressed such as limitations of time and place in accessing healthcare, it can be an important facilitator for disadvantaged groups to engage in help-seeking [66]. It is also a viable strategy that widens the reach and promotes ease of access to intervention, enabling the under-represented population to participate remotely, thus breaking the barriers of distance [67]. This is demonstrated by a study in China which identified factors that could affect the uptake of eMental Health intervention amongst Filipino migrant domestic workers, namely: (1) younger age; (2) longer time as a migrant worker; (3) likely to seek professional services; (4) with financial capacity and willingness to pay for services; (5) belief on mental health services; (6) technological access; and (7) higher levels of social support [68].

Despite the extraordinary circumstance of the Covid-19 lockdown when the intervention was conducted, no drop-out of participants was recorded. The 97 per cent attendance rate in all sessions indicates an excellent uptake of the program and the acceptability and feasibility of the intervention. It takes a great deal of effort to engage migrant domestic workers in an intervention. Constraints such as time, privacy, technological resources, and lack of digital skills and motivation may hamper their participation in an online mode of intervention. As vulnerable groups in society, especially those with limited immigration status and those who are survivors of human trafficking, the delivery of the intervention needs to be done in a conscientious way that ensures safety, as well as offers convenience such as online delivery to encourage participation [69]. The authors did try and ensured the safety and convenience of the participants by signposting them to relevant mental health services and immigration support and providing them with one-on-one assistance in installing and using the Zoom application. Other feasibility problems of the online MHL program include the length of the sessions which participants found challenging. This is consistent with the findings of previous studies that emphasized the importance of shortened intervention among refugees [70] and immigrants [69].

### The potential effectiveness of the culturally adapted online MHL program

This is an early phase of intervention development, and we acknowledge the limitations on measures of its potential effectiveness with a relatively small sample without a control group in the pilot study. As an intervention, psychoeducation gives individuals an opportunity to learn and consider misconceptions about mental health and illness and is shown to improve mental health literacy [71, 72]. In turn, it empowers individuals in taking positive action to improve their mental health, manage their distress, and build resilience. As well as facilitating informed decisions and insight about illnesses such as those related to signs, symptoms, and risk factors associated with common mental disorders, it also helps in gaining an understanding of available treatment and other help-seeking options. It promotes the use of effective self-help strategies and healthy coping behaviors for the prevention and management of psychological distress [30]. It also leads to greater self-awareness in recognizing symptoms, identifying severity levels, and understanding triggers of their psychological distress. This helps facilitate the acknowledgement of mental health issues [73] to gain self-control and manage negative emotions. In a meta-analysis done by Banish and his colleagues [74], a culturally adapted intervention was found to be more effective than unadapted versions of the same intervention. This was supported by the findings of Hall and his colleagues [35] that culturally adapted interventions produce greater reductions in symptomatology than another intervention or no intervention at all. Hence, cultural adaptation makes interventions meaningful and useful to culturally diverse groups for which it was designed.

Studies have shown that mental health literacy promotes help-seeking behavior [32] Given that the major content of this intervention is on normalizing the need for specialist services for mental health, this study demonstrated that a culturally adapted online MHL program can result in positive changes in attitudes toward professional help-seeking. The observed attitudinal changes in participants’ preference from informal help to formal help support this claim. This is also consistent with the assertion that psychoeducation such as this online MHL program can be considered a core element in cultural adaptation because of its potential to cause behavior changes [28]. This is in contrast with the claim of Chu and Leino [75] that psychoeducation should only be treated as a peripheral component of cultural adaptation of an evidence-based intervention.

In a systematic review of experimental studies on cultural adaptation of minimally guided interventions for the treatment of common mental disorders, Shehadeh and colleagues [22] found that intervention effectiveness increases as the number of implemented adaptation elements increase when they are used in diverse settings. Although the current MHL program is still in its feasibility phase, it managed to implement at least 6 out of 8 components of cultural adaptation of intervention identified by Bernal and his colleagues [76], namely: (1) language; (2) metaphors; (3) content of intervention; (4) concept of illness, (5) methods of delivery; and (6) context of the intervention or services. The two other components, therapeutic relationship, and treatment goals, need further adaptation.

The culturally adapted intervention also has the potential to bridge the gap between mental health needs and services [77]**.** Help-seeking in the Philippines was seen as being very different to that in the UK because there are more mental health resources in the latter [78]. Mental health services in the Philippines are less accessible and more expensive, and laden with problems of facilities and staff shortages [79]. Thus, mental ill-health is not taken seriously, unless it is very severe [19]**,** for fear of creating an undue burden on the family already constrained by financial problems [80]. This leads to stigmatizing beliefs and feelings of shame about experiencing mental health problems. Although the results of the qualitative FGD showed a reduction in self-stigma among participants, however, the quantitative changes in stigma tolerance were not maintained at 2 months follow-up, which may suggest the need for a booster session. This is similar to the findings in a study in Ghana on the impact of MHL on stigma tolerance [81] which indicates that a higher threshold level of MHL must be reached before changes in an individual’s indifference to stigma becomes apparent.

Nevertheless, the facilitation of group sharing in which individuals can openly talk about sensitive topics like mental health issues has also been shown to lessen the stigma which is a significant barrier to help-seeking [32]. Some of the helpful processes may have been women talking openly about their experience of mental health problems in the context of a supportive group. This is consistent with the findings in a study on the strong positive correlation between MHL and stigma tolerance among participants [82]. Through group conversations, migrant domestic workers are allowed to talk about their personal experiences where they feel safe and supported without fear of negative judgment from peers. Group conversations with peers served the important function of mutual help groups that validate their experience [62]. It also helps them vent out pent-up emotions that they would not likely share with others if they felt discriminated against or stigmatized**.** Such favorable attitudes may have been reinforced by the modeling of the behavior and attitude of fellow participants. In a scoping review of the literature on peer support for mental health problems of migrant domestic workers, Ho and colleagues [2] suggested that training para-professionals to provide emotional comfort and promote effective coping strategies is an effective approach and culturally appropriate for this subcultural group. Migrant domestic workers commonly seek connection and emotional support from their social networks. Distance and separation from their families and loved ones magnifies their need for emotional support, in which they mostly gravitate towards peers in their host country who share similar beliefs, ethnicity and culture [83].

### Methodological limitations

Several methodological limitations of this pilot study need to be considered. First, the small sample size limits the statistical power to establish significant differences in outcome measures in various testing periods. The homogeneity of the participants in terms of gender and ethnicity and the process of purposive recruitment may pose limitations to the generalizability of the results. The lack of a control group and the absence of randomization further limit the effects of the intervention because it cannot rule out the influence of confounding factors. Another limitation is the use of self-report questionnaires which may have resulted in reporting biases and difficulties reported in answering an online assessment. Although there was no drop-out among participants, the timing of the conduct of the intervention during the Covid-19 lockdown situation when stress is high might had an impact on their retention and understanding of information on mental health. The intervention was also relatively brief and compact with only three sessions of two-hour duration, yet the sessions covered a wide range of information regarding mental health and illness, coping strategies, and help-seeking. It would seem beneficial if the online MHL program was delivered in 5–6 sessions with a shorter time duration each to allow for more in-depth discussion and for participants to digest the key concepts and information. The use of online technology both in the delivery of the intervention and in the conduct of the assessment may also pose some limitations because not all participants are adept at using remote learning tools. Their test performance may have been affected by their frustrations in taking online assessments when they encountered technical difficulties.

Despite several limitations of this study, the findings contribute to the current literature on culturally adapted online MHL programs specially designed for an under-represented population like migrant domestic workers. However, the same approach in intervention development in the future can also be used in other migrant populations in the UK. The MHL program was also delivered in a community-based setting and thus may contribute to the stepped-care model developed by the United Nations’ Inter-Agency Standing Committee (IASC) [84]. Given the limitations of this pilot study, further evaluation using another feasibility trial is needed before a randomized controlled trial for the efficacy test of the current intervention is undertaken. This will allow for the refinement of the intervention development into a more practical, acceptable, and effective means of intervention for an under-represented population like migrant domestic workers.

## Conclusion

To the best of our knowledge, this is the first pilot study that investigates the potential effectiveness, acceptability, appropriateness, and feasibility of a culturally adapted MHL program that is delivered entirely online for Filipino migrant domestic workers in the UK. Quantitative findings indicated a promising potential for its effectiveness in improving mental health literacy and help-seeking propensity. This was supported by qualitative data from the FGDs in which participants reported changes in their understanding of mental health and symptoms of common mental disorders and having positive attitudes towards formal help-seeking. Its cultural adaptation integrating Filipino cultural concepts of mental health and well-being, illustrative case examples, and use of local language and Filipino metaphors, is acceptable and feasible for use among Filipino migrant domestic workers. Although certain methodological limitations are observed, the content, form, and delivery of the intervention were deemed relevant and appropriate to the needs and situation of this under-represented group. Given their vulnerable position in society in which they are prone to isolation, loneliness, anxiety, abuse, exploitation, and trauma, yet are less likely to seek professional help, this online MHL program has a clinical promise of providing support to their mental health and well-being. A large-scale randomized controlled trial is needed to confirm the preliminary findings of this study.

## Data Availability

The data that support the findings of this study are available on request from the corresponding author. The data are not publicly available due to privacy or ethical restrictions.

## Appendix 1: Case Study (Identifying symptoms of depression)

*Si Mariah ay 45 na taong gulang, hiwalay sa asawa at may dalawang anak. Dumating siya sa UK noong 2005 bilang estudyante ngunit nagsara ang kanyang unibersidad at napilitan siyang magtrabaho dahil sa laki ng iniwang utang sa Pilipinas. Naging undocumented migrant, pinili niyang manatili at magtrabaho sa UK para mapag-aral ang mga anak at mabayaran ang kanilang utang. Hindi nakakauwi si Mariah sa Pilipinas kahit para madalaw man lang ang kanyang mga anak na ngayon ay mga binata at dalaga na, maging ang kanyang mga magulang na ngayon ay mahina at matatanda na.*

[Mariah is 45 years old, separated from her husband and has two children. She came to the UK in 2005 as a student but her university closed, so she was forced to find work because of the amount of debt she left behind in the Philippines. Having become an undocumented migrant, she chose to stay and work in the UK so she can send her children to school and pay off her debt. Mariah is unable to return to the Philippines even just to visit her children who are now grown-ups, and her parents, who are now elderly and very frail.]

*Noong unang lockdown, nawalan ng trabaho si Mariah dahil namatay ang matandang Briton na kanyang inaalagaan nang limang taon. Naiwang nag-iisa at hindi makalabas ng bahay dahil sa lockdown, nagsimulang makaramdam si Mariah ng matinding lungkot. Idagdag pa sa kanyang pinagdadaanan, inatake sa puso at namatay ang kanyang ama na naiwan sa Pilipinas at matagal na niyang hindi nakikita ng 16 taon. Dahil sa pandemic, hindi siya makauwi sa Pilipinas at hindi rin naiburol nang matagal ang kanyang ama upang makapagluksa sila.*

[During the first lockdown, Mariah lost her job because the elderly British employer she had been caring for 5 years passed away. Left alone and isolated due to lockdown, Mariah began to feel deeply depressed. In addition to what she was going through, her father who was left behind in the Philippines and whom she had not seen for 16 years had a massive heart attack and died. Due to the pandemic, she cannot come home to the Philippines and her family back home could not have a longer funeral for them to mourn the passing of their father].

*Dahil sa mga pangyayari, madalas ay hindi siya makakain at makatulog nang maayos. Sobra ang naging pag-aalala nya sa baka magkasakit siya ng Covid-19 at kadalasan na naiisip niya na mag-isa siyang mamamatay. Ang dating masayahin at makwentong si Mariah ay unti-unting naging tahimik at nawalan nang ganang makipag-usap sa mga kaibigan.*

[Due to the circumstances, she often could not eat and had trouble sleeping well. She was extremely worried that she might get infected with Covid-19 and often thought she would die alone. The once cheerful, bubbly and chatty Mariah gradually became very sullen and withdrawn and lost her desire to talk to friends].

*Unti-unting bumabagsak ang kanyang katawan at nakakalimot na siyang alagaan ang sarili. Ang dating masigla at malinis sa katawan na Mariah ay unti-unting napabayaan ang sarili, may mga araw pa nga na hindi na siya naliligo.*

[Little by little, her body is failing and she is forgetting to take care of herself. The once energetic and very hygienic Mariah has gradually neglected herself; there are even days when she doesn’t take a shower anymore.]

*Madalas siyang nakatulala, at umiiyak. Pakiramdam ni Mariah ay lagi siyang pagod at wala nang saysay ang kanyang buhay. Sinisisi niya ang kanyang sarili sa pagkamatay ng ama at ng matandang Briton na kanyang alaga.*

[She was often immovable, and crying. Mariah feels that she is always exhausted and that her life no longer makes sense. She blames herself for the death of her father and the old Briton she cared for].

*Anu-ano ang mga sintomas ng problema sa kalusugan ng isip na inyong makikita kay Mariah?*

[What are the indications of mental health problems that you have seen in Mariah?].

*Kung ikaw ang nasa sitwasyon ni Mariah, ano ang iyong maaaring gawin upang tugunan ang iyong pinagdadaanan?*

[If you are in Mariah’s situation, what could you do to address your problems?].
